# Hierarchical Design of CuO/Nickel–Cobalt–Sulfide Electrode by a Facile Two-Step Potentiostatic Deposition

**DOI:** 10.3390/mi14040888

**Published:** 2023-04-20

**Authors:** Sa Lv, Peiyu Geng, Yaodan Chi, Huan Wang, Xuefeng Chu, Yang Zhao, Boqi Wu, Wenshi Shang, Chao Wang, Jia Yang, Zhifei Cheng, Xiaotian Yang

**Affiliations:** Key Laboratory for Comprehensive Energy Saving of Cold Regions Architecture of Ministry of Education, Jilin Jianzhu University, Changchun 130118, China

**Keywords:** electrode material, transition metal sulfides, nanosheet, potentiostatic deposition

## Abstract

Herein, a scalable electrodeposition strategy is proposed to achieve hierarchical CuO/nickel–cobalt–sulfide (NCS) electrodes using two-step potentiostatic deposition followed by high-temperature calcination. The introduction of CuO provides support for the further deposition of NSC to ensure a high load of active electrode materials, thus generating more abundant active electrochemical sites. Meanwhile, dense deposited NSC nanosheets are connected to each other to form many chambers. Such a hierarchical electrode prompts a smooth and orderly transmission channel for electron transport, and reserves space for possible volume expansion during the electrochemical test process. As a result, the CuO/NCS electrode exhibits superior specific capacitance (*C*s) of 4.26 F cm^−2^ at 20 mA cm^−2^ and remarkable coulombic efficiency of 96.37%. Furthermore, the cycle stability of the CuO/NCS electrode remains at 83.05% within 5000 cycles. The multistep electrodeposition strategy provides a basis and reference for the rational design of hierarchical electrodes to be applied in the field of energy storage.

## 1. Introduction

Electrode materials, as the core components of energy storage devices, have continuously received extensive attention from researchers [1]. Among the various types of electrode materials available, transition metal sulfides, particularly Ni/Co sulfides and including Ni-Co bimetallic sulfides, have received tremendous attention for their outstanding merits of high theoretical specific capacitance (*C*s), multiple oxidation states, and the lower electronegativity of sulfur compared to oxide counterparts [2,3,4]. For example, Meng et al. reported Ni-Co-S (NCS) nanoarchitecture on nickel foam via the combination of hydrothermal and sulfurization processes. This in situ growth accelerated the rapid transport of ions/electrons, and the porosity of NCS nanosheets allowed for fuller contact with the electrolyte; thus, the optimized electrode *C*s reached 6.3 F cm^−2^ at 1 mA cm^−2^ [5]. In addition, the researchers further improved the *C*s and cycling performance of NCS electrodes by exploring various synthetic pathways to regulate the quality of the growing active materials [6,7,8,9]. Among them, electrodeposition has proven to be an effective strategy which can adjust the amount of deposition layer by changing the electrolyte concentration, deposition time, and other parameters to avoid the structural collapse caused by excessive deposition and shedding during the charge–discharge process. For instance, Wen et al. developed hierarchical NCS electrodes by electrodeposition. The effects of metal salt solution concentration and deposition time on load quality were investigated in detail. The *C*s attenuation of the optimized electrode for 3000 cycles was only 5% at a current density of 10 A g^−1^ [10]. At the same time, the researchers also explored the construction of composite electrodes based on NCS to fully take advantage of their synergistic effect. For example, Wang et al. designed the NCS/carbon nanotube composite electrode, which achieved an excellent *C*s of 8.62 F cm^−2^ at 5 mA cm^−2^. The selection of carbon nanotube components stems from their high conductivity and provides support for the deposition of NCS, thus forming more abundant electrochemically active sites [11]. As another example, Ma et al. adopted a similar strategy to conceive hierarchical CoMoS_4_@NCS electrodes on carbon cloth, and systematically evaluated the influence of NCS electrodeposition quality on overall performance. The optimized CoMoS_4_@NCS electrode exhibited ultra-high *C*s and desirable cycling stability [12]. Therefore, electrodeposition is reasonably considered a preferred approach to achieve hierarchical stepwise growth and precise regulation of the amount of deposition layers. In addition, it also reflects the advantages of short experiment cycles, high repeatability, and convenient operation [13].

Inspired by this, in this paper, we designed and constructed hierarchical CuO/NCS electrodes on copper foam (CF) by a facile two-step potentiostatic electrodeposition and intermediate calcination process. The resulting NCS nanosheets were tightly wrapped around bent CuO nanorods. This hierarchical rational design not only created abundant electrochemical active sites, but also facilitated ion/electron transmission and accelerated efficient pseudocapacitance reactions, thus exhibiting excellent electrochemical activity. 

## 2. Experimental Section

### 2.1. Materials

CF (1 cm × 1.5 cm) was cut in advance; treated with ethanol, dilute hydrochloric acid, and water to remove surface impurities; and, finally, vacuum-dried.

### 2.2. Potentiostatic Deposition of CuO/NCS on CF

A three-electrode system consisting of CF (working electrode), Pt foil (counter electrode), and Hg/HgO (reference electrode) performed the operation in 2 M NaOH. After 300 s of potentiostatic deposition (−0.1 V), orange-red CF was etched and oxidized to produce blue-green Cu(OH)_2_, and then calcined at 200 °C for 2 h to form black CuO. 

A three-electrode system consisting of CuO was obtained in the previous step as the working electrode, with Pt foil and SCE as the counter electrode and reference electrode, respectively. The electrolyte contained 0.05 M NiCl_2_·6H_2_O, 0.05 M Co(NO_3_)_2_·6H_2_O, and 0.5 M CH_4_N_2_S. After 300 s of potentiostatic deposition (−1.1 V), the CuO/NCS composite electrode was thus obtained. Additionally, the deposition time of the NCS layer was also set at 30 s, 150 s, and 450 s for comparison.

### 2.3. Characterization 

The morphology and structure of the products were characterized, including X-ray diffraction (XRD), scanning electron microscopy (SEM), and X-ray photoelectron spectroscopy (XPS). At the same time, an electrochemical workstation (760 E) was used to test the cyclic voltammetry (CV), galvanostatic charge–discharge (GCD), electrochemical impedance spectroscopy (EIS), and cycle stability of the CuO/NCS electrode. A three-electrode system consisting of CuO/NCS (working electrode), Pt foil (counter electrode), and Ag/AgCl (reference electrode) was tested in 2 M NaOH.

## 3. Results

**Figure 1:**
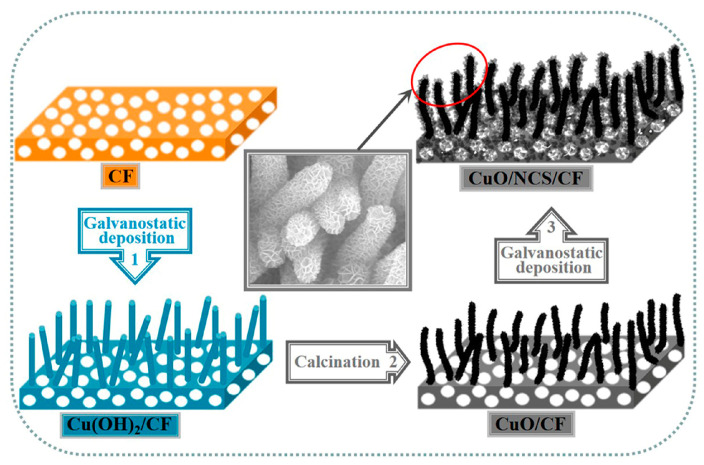
The design process of CuO/NCS electrode.

Figure 1 depicts the design process of the CuO/NCS composite electrode. First, CF with a typical 3D structure was selected as the electrode substrate, as it has good conductivity and can provide a large specific surface area for the deposition of active electrode materials. Enlarged images confirmed that the surface of the CF filament was relatively flat (Figure S1). CF was oxidized and released Cu^2+^, which was captured by OH^−^ to form blue-green Cu(OH)_2_ nanorod arrays by potentiostatic deposition technology (Step 1), and then calcined into black, slightly bent CuO (Step 2). CF plays a dual role as an electrode substrate and copper source. After this, potentiostatic deposition was performed again to place the coating of the NCS layer onto the surface of CuO. The final formation of the CuO/NCS electrode was achieved (Step 3). Thiourea provided the sulfur source for NCS, and its hydrolysis reaction was determined by Equation (1); it could also be explained by the fact that thiourea directly generates S^2−^ in an alkaline medium (Equation (2)) [10]. Furthermore, the synthesis reaction of metal sulfides (MS, M = Ni/Co) could be expressed as Equation (3):

(1) $${\left(NH_{2}\right)}_{2}CS+H_{2}O\to H_{2}S+CO_{2}+2NH_{3}$$

(2) $${\left(NH_{2}\right)}_{2}CS+2OH^{-}\to H_{2}NCN+S^{2-}+2H_{2}O$$

(3) $$M^{2+}+S^{2-}\to MS$$

**Figure 2:**
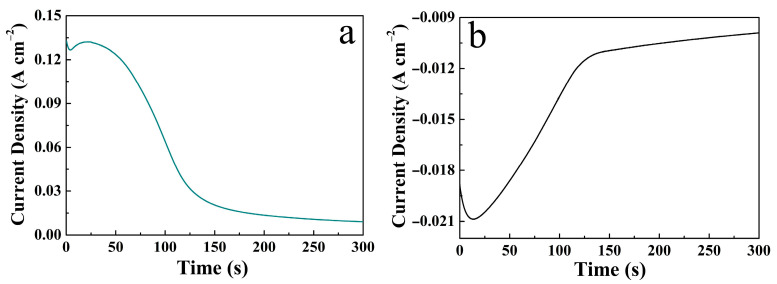
Two-step potentiostatic deposition curves of the CuO/NCS electrode: (**a**) Cu(OH)_2_ deposited onto the CF substrate; (**b**) NCS deposited onto the CuO surface.

That is, in the initial stage of electrodeposition, the S^2−^ was generated on the surface of the CuO electrode by hydrolysis of thiourea, which was captured by the Ni^2+^/Co^2+^ to form NCS nuclei, and then gradually grew into NCS nanosheets due to their preferred growth surface. The two-step potentiostatic deposition curves are shown in Figure 2.

**Figure 3:**
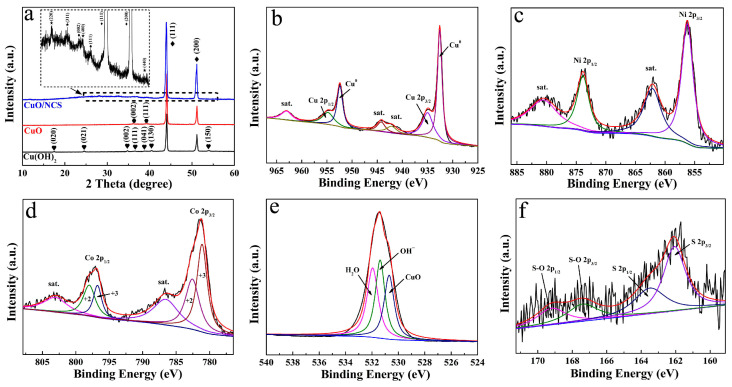
XRD patterns of Cu(OH)_2_, CuO, and CuO/NCS (**a**). XPS spectra of (**b**) Cu 2p, (**c**) Ni 2p, (**d**) Co 2p, (**e**) O 1s, and (**f**) S 2p for CuO/NCS.

Furthermore, the composition and structure of the CuO/NCS electrode were analyzed in detail. Figure 3a records the XRD pattern of Cu(OH)_2_, CuO, and CuO/NCS. Among them, the two strong peaks marked with rhombuses came from CF substrate (JCPDS 01-1241). The red line represents CuO [JCPDS 05-0661], which was oxidized from Cu(OH)_2_ (JCPDS 13-0420). However, the diffraction peaks of CuO did not change significantly after the deposition of the NCS layer due to the strong contrast of the CF substrate [12]. As a result, the diffraction peaks were locally amplified as the insertions, and the diffraction peaks marked with stars came from NCS (JCPDS no. 43-1477). In addition, the composition of Cu, Ni, Co, O, and S elements in CuO/NCS was verified by XPS spectra. Figure 3b shows the Cu 2p spectrum, deconvoluted into two prominent peaks at 954.50 and 934.60 eV, corresponding to Cu 2p_1/2_ and Cu 2p_3/2_, and the two sharp peaks at 952.45 and 932.55 eV originated from the CF substrate [14,15]. The other three peaks at 963.03, 944.80, and 941.90 eV belonged to satellite peaks. The Ni 2p spectrum is exhibited in Figure 3c. A pair of spin-orbit doublets, Ni 2p_1/2_ and Ni 2p_3/2_, at 873.85 and 856.23 eV belonged to Ni^2+^, and their corresponding satellite peaks appeared at 880.50 and 861.80 eV, which was consistent with previous work [16,17]. As illustrated in Figure 3d, the deconvolution of the Co 2p profile manifested two sets of peaks. Among them, the two peaks at 798.00 and 796.50 eV corresponded to Co2p_1/2_, while 782.60 and 781.10 eV corresponded to Co2p_3/2_, indicating the coexistence of Co^2+^ and Co^3+^ [12,18]. Additionally, the other two peaks at 803.05 and 786.85 eV were assigned to satellite peaks. Figure 3e shows the O 1s spectrum; three peaks, located at 532.20, 531.40, and 530.70 eV, were ascribed to H_2_O, OH^−^, and CuO [19]. From the S 2p spectrum in Figure 3f, these can be resolved into two S species. The peaks at 163.50 and 162.07 eV, corresponding to S 2p_1/2_ and S 2p_3/2_, could be ascribed to S^2−^ [4,11], while the two weak peaks at 169.20 and 167.40 eV corresponded to S-O 2p_1/2_ and S-O 2p_1/2_, respectively [20,21]. Oxygen came from OH^−^, produced by the hydrolysis of thiourea, which was consistent with previous reports [10].

**Figure 4:**
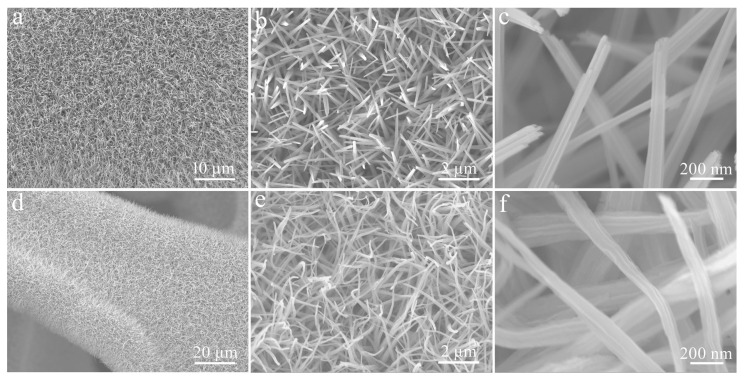
SEM images of (**a**–**c**) Cu(OH)_2_ and (**d**–**f**) CuO.

Figure 4 depicts SEM images of Cu(OH)_2_ and CuO, obtained by calcination at different magnifications. As shown in Figure 4a, the surface of CF was covered by a uniform and dense Cu(OH)_2_ layer. The corresponding magnified images confirmed that these Cu(OH)_2_ had straight, rod-like structures and smooth surfaces circa 115 nm in diameter (Figure 4b,c). The CuO produced by calcining Cu(OH)_2_ nanorods at 200 °C still retained the 3D structure of CF (Figure 4d), but these nanorods became bent due to water loss, and the diameters of the nanorods did not change (Figure 4e,f). 

**Figure 5:**
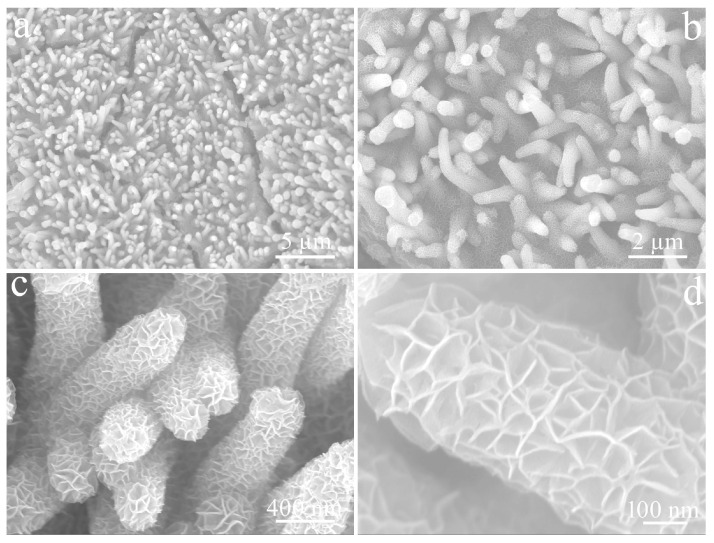
Low (**a**,**b**) and high (**c**,**d**) magnifification SEM images of CuO/NCS composite (with NCS layer deposited for 300 s).

Figure 5 reveals the SEM images of CuO/NCS at different magnifications. As shown in Figure 5a, the overall distribution remained uniform and orderly, except for fine cracks on the sample’s surface. Compared to bent CuO nanorods, the CuO/NCS rod-like structure became relatively robust and the orderliness significantly improved (Figure 5b). That is because the NCS layer was uniformly deposited on the surface of CuO nanorods, which made the rod-like structure more compact and increases the diameter to circa 430 nm (Figure 5c). After further amplification, it could be seen that what were tightly wrapped around the surface of CuO nanorods were NCS nanosheets with a thickness of circa 6 nm, and these curved nanosheets were closely interwoven with each other to form many chambers (Figure 5d).

**Figure 6:**
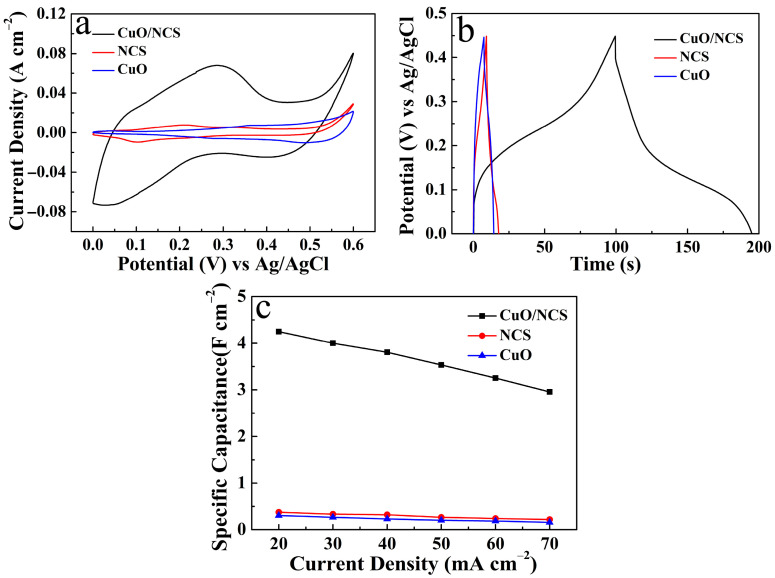
Comparison of energy storage properties of the CuO/NCS, NCS, and CuO electrodes: (**a**) CV curves; (**b**) GCD curves; (**c**) *C*s at different current densities.

The electrochemical properties of the products were tested and analyzed. Figure 6a shows the CV curves of the CuO/NCS, NCS, and CuO electrodes at a scan rate of 10 mV s^−1^. We followed the principle that the larger the area of the CV curve was, the larger the *C*s would be [10,22]. The CuO/NCS electrode had the largest *C*s of the three electrodes. This trend is also illustrated in Figure 6b. When the discharge current density was set to 20 mA cm^−2^, the CuO/NCS electrode exhibited the longest discharge time compared to the NCS and CuO electrodes, and, thus, has the largest *C*s [4]. The corresponding relationship between the current density and the *C*s of the three electrodes is shown in Figure 6c. The significantly enhanced energy storage characteristics of the CuO/NCS electrode were attributed to the following pseudocapacitance reactions [12,17,23]:

(4) $$NiS\;+\;OH^{-}↔NiSOH\;+\;e^{-}$$

(5) $$CoS\;+\;OH^{-}↔CoSOH\;+\;e^{-}$$

(6) $$CoSOH\;+\;OH^{-}↔CoSO\;+\;H_{2}O\;+\;e^{-}$$

**Figure 7:**
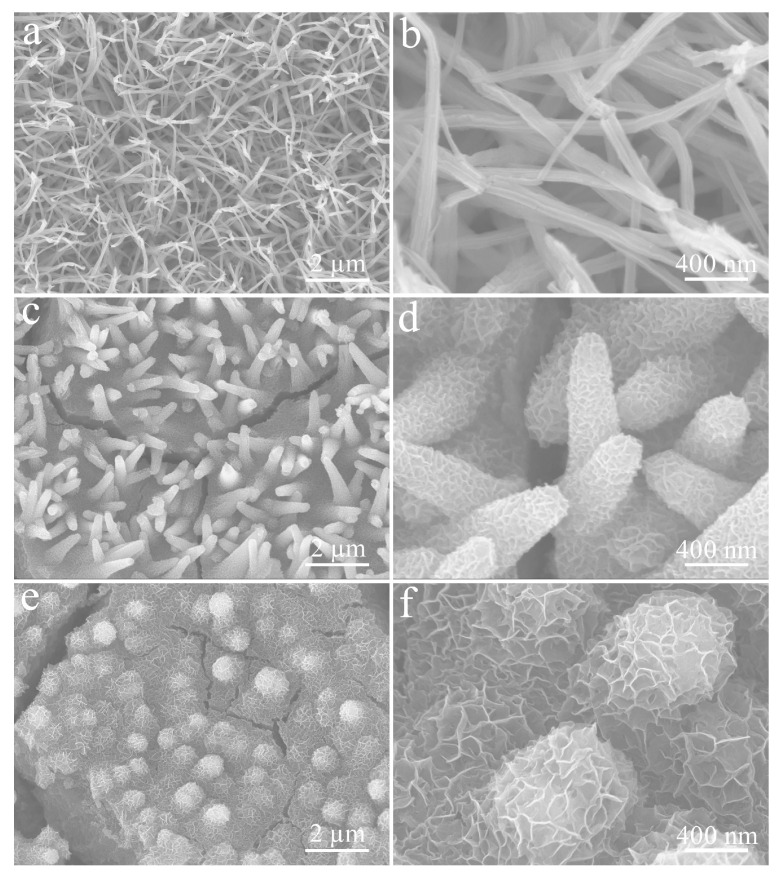
SEM images of CuO/NCS composites with different NCS deposition time: (**a**,**b**) 30 s; (**c**,**d**) 150 s; (**e**,**f**) 450 s.

We also regulated the deposition time of the NCS layer. At the initial deposition stage (30 s), the curved, rod-like CuO had little change, but the surfaces of the nanorods began to become rough (Figure 7a,b). When the NCS deposition time was extended to 150 s, as shown in Figure 7c, these nanorods gradually became thick and distinct due to being tightly wrapped by dense, wrinkled, growing NCS nanosheets (Figure 7d). When deposition was carried out for 300 s, the sizes of these NCS nanosheets gradually increased and became denser, as previously described in Figure 5. Continuous excessive deposition (450 s) resulted in the disordered accumulation of a large number of NCS nanosheets, and the rod-like structure of CuO was almost submerged (Figure 7e,f). 

**Figure 8:**
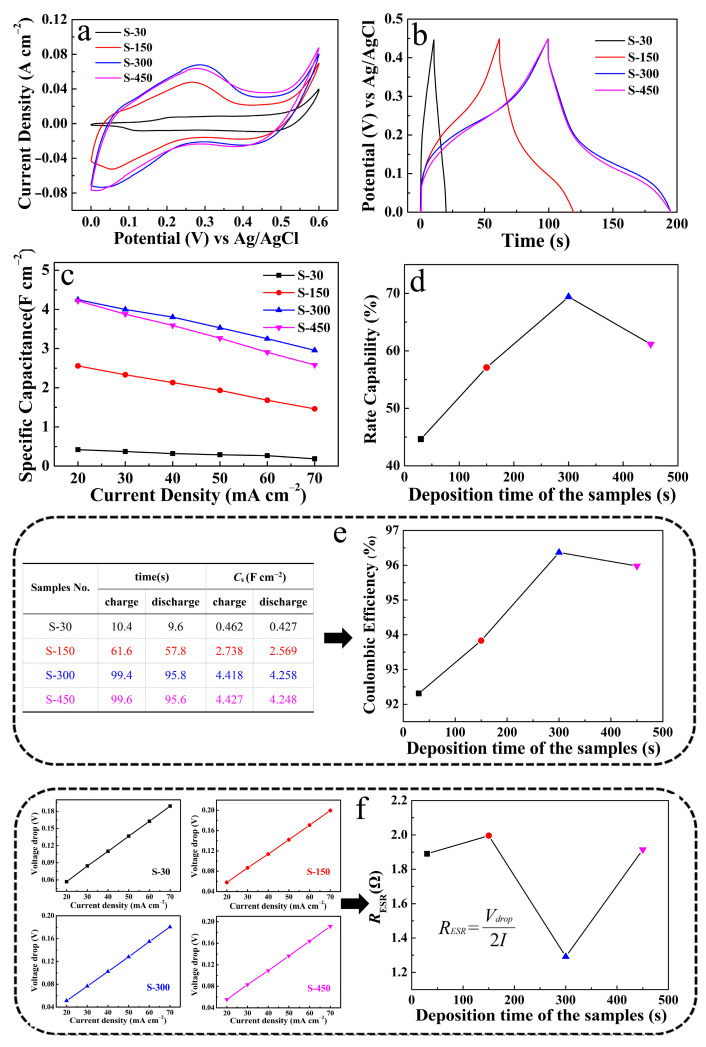
Comparison of energy storage properties of CuO/NCS electrodes with different NCS deposition times (S-30, S-150, S-300, and S-450): (**a**) CV curves; (**b**) GCD curves; (**c**) *C*s at different current densities; (**d**) rate capability; (**e**) coulombic efficiency; and (**f**) average *R*_ESR_.

Based on this, we systematically compared and analyzed the electrochemical performance of the CuO/NCS electrodes with different NCS deposition times (S-30, S-150, S-300, and S-450). Figure 8a records the CV curves of the four electrodes at a scan rate of 10 mV s^−1^. At the initial NCS deposition stage, the energy storage capacity of the electrodes gradually increased with the deposited NCS until the area covered by CV curves at S-300 and S-450 was almost the same. The GCD curves of the four electrodes also reflect the same trend (discharge current density of 20 mA cm^−2^), and the discharge times of S-300 and S-450 were almost the same (Figure 8b). For a more accurate comparison, the specific *C*s values of the four electrodes at different discharge current densities were calculated and listed in Figure 8c. It should be noted that at a current density of 20 mA cm^−2^, the *C*s of S-300 was 4.26 F cm^−2^ and that of S-450 was 4.25 F cm^−2^. Therefore, it was obvious that the order of the four electrodes in terms of energy storage capacity was S-300 > S-450 > S-150 > S-30. Moreover, S-300 maintained 69.41% of the original *C*s when the current density increased from 20 mA cm^−2^ to 70 mA cm^−2^, reflecting the best rate capability (Figure 8d). At the same time, according to the table data (charge and discharge time; *C*s of four electrodes at a current density of 20 mA cm^−2^) on the left in Figure 8e, the coulombic efficiency of S-300 reached as high as 96.37%, which is still the maximum of the four electrodes. In addition, the average *R*_ESR_ data of the four electrodes were compared. The voltage drop data of the four electrodes were substituted into the formula embedded in Figure 8f for calculation, and the *R*_ESR_ of S-300 was 1.29 Ω cm^−2^, the lowest point in the line graph. Therefore, in our experimental system, CuO provided skeleton support and the *C*s of the CuO/NCS electrode gradually increased with the deposited NCS. However, excessive deposition of NCS inevitably led to disordered aggregation and accumulation of the nanosheet structures, which destroyed the supporting role of CuO and was capable of causing collapse and even shedding of the hierarchical structure during the electrochemical test [24]. Therefore, it is necessary to effectively control the deposition time of NCS to give full play to the supporting role of CuO, and, thus, to form CuO/NCS with optimized characteristics. Based on the comparison and analysis of the above data, S-300 was selected for further research.

**Figure 9:**
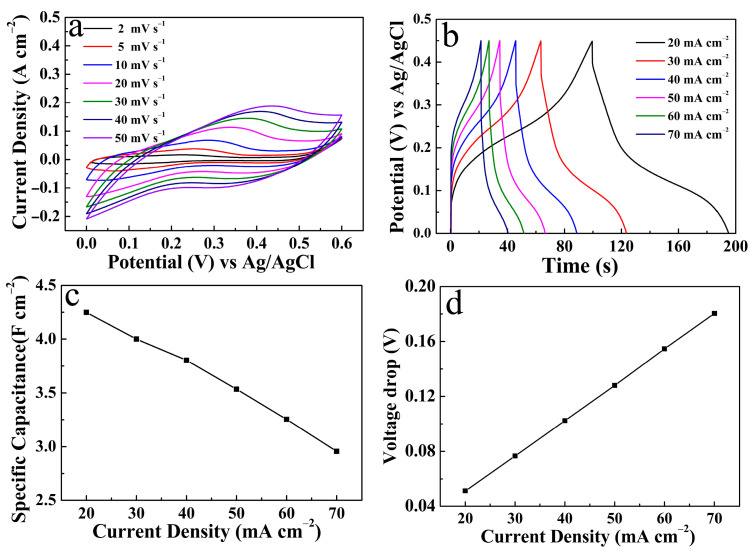
Electrochemical properties of the CuO/NCS electrode (S-300): (**a**) CV curves; (**b**) GCD curves; (**c**) *C*s; and (**d**) voltage drop at different current densities.

Figure 9a shows the CV curve of the CuO/NCS electrode (S-300). As the scan rate increased, the redox peak showed a gradually enhanced current response, while the corresponding *C*s value gradually decreased due to the lack of a sufficient redox reaction between OH^−^ and the electrochemically active site inside the electrode at a relatively large scan rate [22,25]. Figure 9b displays the GCD curve of the CuO/NCS electrode at different discharge current densities. The discharge time of the electrode decreased gradually with the increase in current density, and then the *C*s decreased gradually. According to the formula provided in Supplementary Materials [11], when the current density was 20, 30, 40, 50, 60, and 70 mA cm^−2^, the corresponding *C*s were 4.26, 4.00, 3.80, 3.53, 3.25, and 2.96 F cm^−2^, respectively. The specific correspondences are shown in Figure 9c. The voltage drop data in Figure 9d were described in the previous paragraph. The EIS spectra of the CuO/NCS and NCS electrodes are shown in Figure S2. The Nyquist plots were measured in a frequency ranging of 0.01 Hz–100 kHz. The equivalent serious resistance (*R*s) was equivalent to the point intercepting with the X axis [12,22]; thus, the *R*s values of CuO/NCS and NCS corresponded to 1.09 Ω and 1.13 Ω, respectively. Thereby, the introduction of CuO reduced the internal series resistance, favoring the ions’ diffusion through the electrode/electrolyte [11].

**Figure 10:**
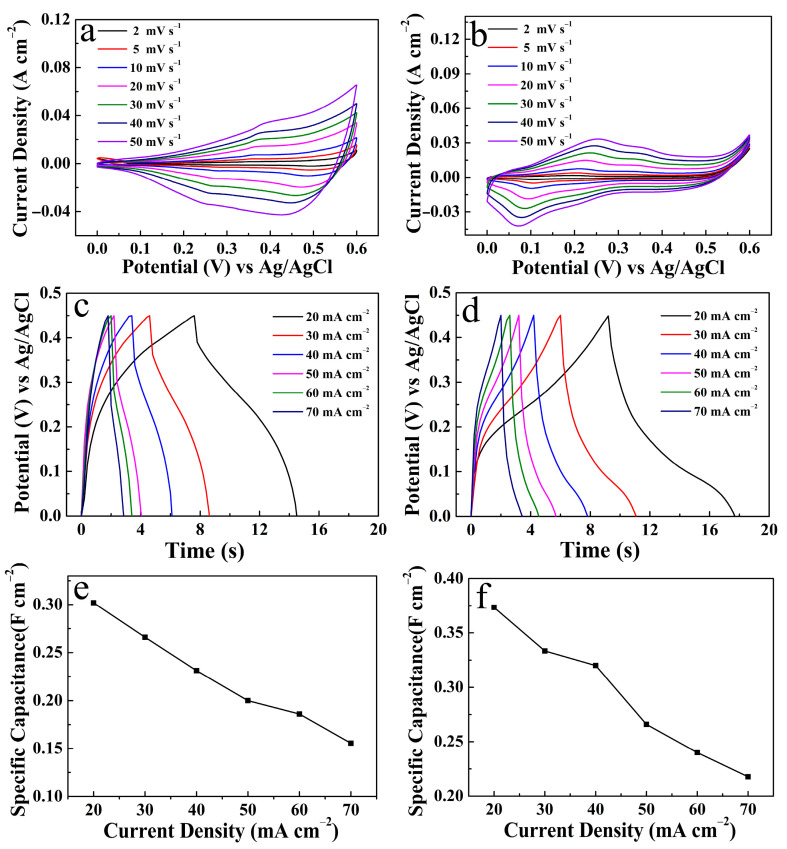
Electrochemical properties of CuO and NCS electrodes: (**a**,**b**) CV curves; (**c**,**d**) GCD curves; and (**e**,**f**) *C*s at different current densities.

**Figure 11:**
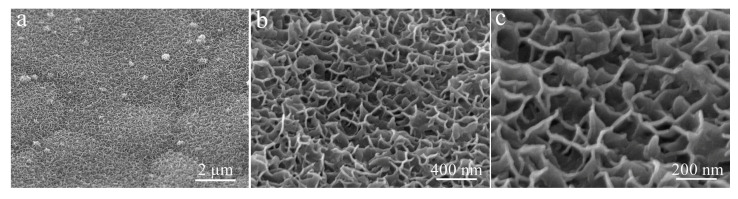
Low (**a**) and high (**b**,**c**) magnifification SEM images of NCS grown on CF at different magnifications.

In addition, the electrochemical properties of CuO and NCS electrodes, including CV, GCD, and *C*s at different current densities, are also recorded in Figure 10. At the same time, it can be seen from Figure 11 that, in the absence of CuO nanorods support, irregular NCS nanosheets were bent and scattered on the surface of the CF substrate. 

**Figure 12:**
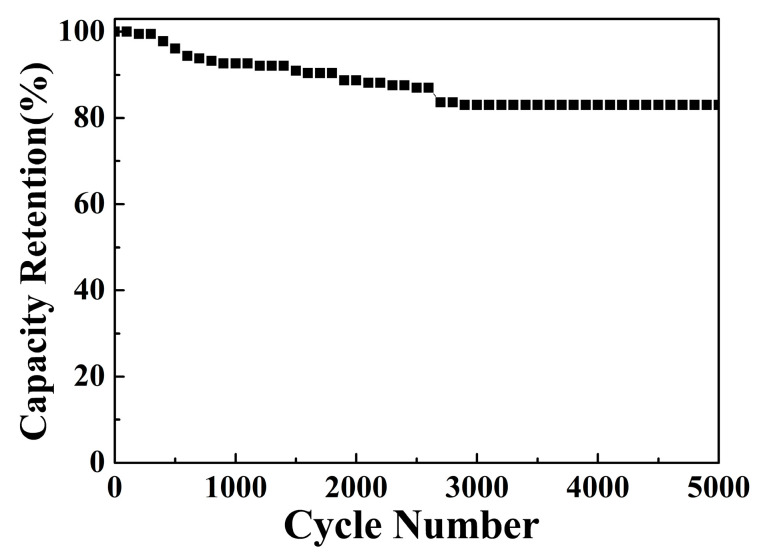
Cyclic stability of the CuO/NCS electrode.

**Figure 13:**
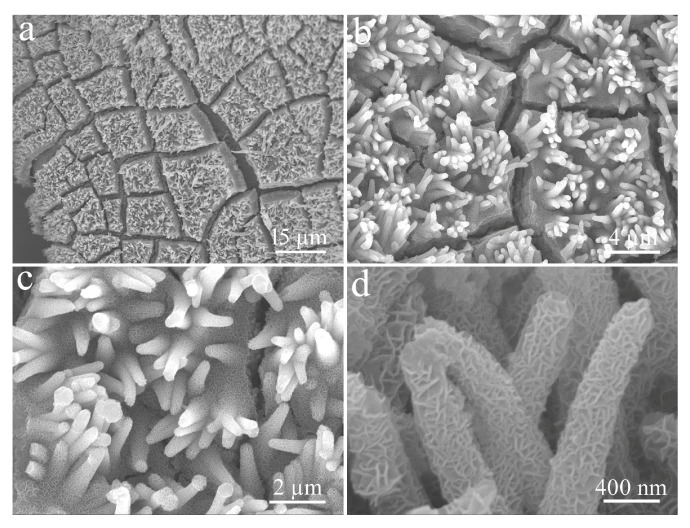
Low (**a**,**b**) and high (**c**,**d**) magnifification SEM images of the CuO/NCS composite after the electrochemical performance test.

Finally, the cyclic stability of the CuO/NCS electrode was tested in Figure 12. The result confirmed that the electrode *C*s remained at 90.96% of the initial value during the first 1500 cycles, and then stabilized at 83.05% within 5000 cycles. In addition, the structure and morphology of the sample after electrochemical performance tests showed little change, except for obvious cracks (Figure 13).

Table 1 compares the test conditions and *C*s values of the relevant electrode materials. The CuO/Ni-Co-S electrode prepared in this work showed relatively prominent energy storage behavior, and the reasons can be summarized as follows: In our reaction system, the choice of CF electrode substrate exhibited direct oxidative corrosion, generating Cu(OH)_2_ nanorods and then forming CuO through subsequent high-temperature treatment. CuO provided support for the further deposition of NSC to ensure high loads of active electrode materials, thus generating more abundant electrochemically active sites [26,27]. These preparation processes can be completed independently via the electrochemical workstation in order to execute the electrodeposition strategy, including subsequent electrochemical performance tests. This fully demonstrates the convenience of the electrochemical method [13,28,29]. In addition, the compact and curved deposited NCS nanosheets are interwoven and tightly wrapped around the CuO nanorod array. This hierarchical CuO/NCS design not only provides a smooth and orderly transmission channel for electron transport [30], but also reserves space for possible volume expansion during the electrochemical test process while also ensuring structural stability during the long-term electrochemical performance test [4,11,19,31].

## 4. Conclusions

In summary, hierarchical CuO/NCS electrodes were uniformly grown on CF substrates by two-step potentiostatic deposition. As one of the active components, CuO also provided spatial support for NSC deposition. Meanwhile, the effects of the NCS deposition time on the energy storage characteristics of CuO/NCS electrodes were systematically compared and analyzed. The optimized CuO/NCS electrode *C*s reached 4.26 F cm^−2^ at a current density of 20 mA cm^−2^, and the coulombic efficiency reached as high as 96.37%. At the same time, its *C*s remained at 85.03% of the initial value within 5000 cycles. This multistep electrodeposition strategy can be extended in order to design other CuO-based binary or multicomponent transition metal sulfide electrodes to meet the high performance requirement of electrochemical energy storage devices.

## Figures and Tables

**Table 1:** *C*s evaluation of relevant electrode materials.

Electrode	Substrate	Electrolyte	Current Density (mA cm^−2^)	*C*s (F cm^−2^)	Ref.
CuO	Ni foam	6 M KOH	3	1.61	[32]
CuO	brass	1 M Na_2_SO_4_	5	1.68	[33]
Ni(OH)_2_@CuO	Carbon cloth	6 M KOH	1	2.28	[34]
CuO/ppy	Cu foam	3 M KOH	2	1.08	[35]
Ni-Co-S	Ni foam	3 M NaOH	10	4.87	[36]
Ni-Co-S	Ni foam	1 M KOH	1	3.31	[37]
Co-Ni-S	Ni foam	3 M KOH	8	1.12	[5]
Se-Ni-Co-S	Ni foam	3 M KOH	1	2.00	[38]
CuO/Ni-Co-S	Cu foam	2 M NaOH	20	4.26	this work

## Data Availability

Not applicable.

## Supplementary Materials

The following are available online at https://www.mdpi.com/article/10.3390/mi14040888/s1, Figure S1: SEM images of bare CF at different magnifications. Figure S2: EIS spectra for CuO/NCS and NCS electrodes. Formula for calculating the *C*s.
