# Supplementary material for: Hierarchical Design of CuO/Nickel–Cobalt–Sulfide Electrode by a Facile Two-Step Potentiostatic Deposition
Source: Micromachines (Basel). 2023 Apr 20;14(4):888. doi: 10.3390/mi14040888 (PMC10143578; doi:10.3390/mi14040888)
Supplement: Supplementary file 1 [file micromachines-14-00888-s001.zip › micromachines-2278451-supplementary.pdf]

Supplementary Materials

Hierarchical Design of CuO/Nickel-Cobalt-Sulfide Electrode by a Facile Two-Step Potentiostatic Deposition

Sa Lv *, Peiyu Geng, Yaodan Chi, Huan Wang, Xuefeng Chu, Yang zhao, Boqi Wu, Wenshi Shang, Chao Wang, Jia Yang, Zhifei Cheng and Xiaotian Yang *

Key Laboratory for Comprehensive Energy Saving of Cold Regions Architecture of Ministry of Education, Jilin Jianzhu University, Changchun 130118, China; gengpeiyu 1@163.com (P.G.); chiyaodan@jlju.edu.cn (Y.C.); wanghuan@jlju.edu.cn (H.W.); stone2009@126.com (X.C.); zhaoy261@163.com (Z.Y.); xiancaitang@sina.com (B.W.); shangws0426@163.com (W.S.); wangchao@jlju.edu.cn (C.W.); yangjia@jlju.edu.cn (J.Y.); chengzf838@163. com (Z.C.)

* Correspondence: lvsa@jlju.edu.cn (S.L.); hanyxt@163.com (X.Y.); Tel.: +86-0431-8456-6181 (S.L.)

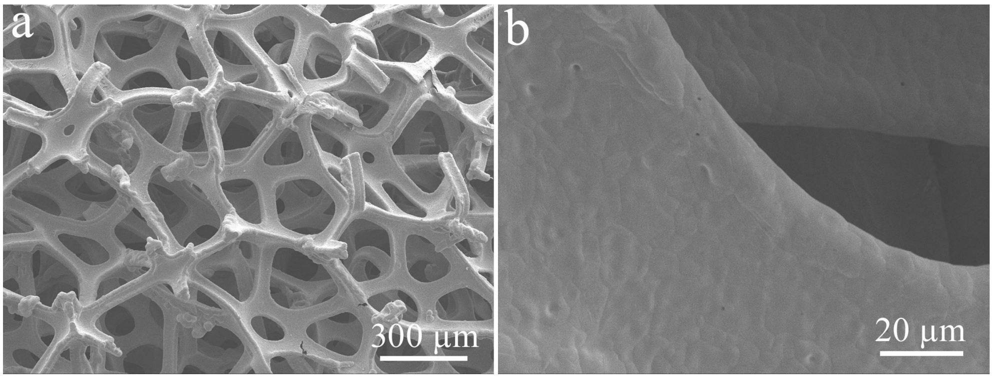

Figure S1. SEM images of bare CF at different magnifications.

The Cs value of CuO/NCS electrode is calculated according to the equation:

$$C_S = \frac{I \times \Delta t}{S \Delta V}$$

where Cs (F cm^{-2}) is the specific capacitance, I (A) is the charge and discharge current, Δt (s) is the discharging time, S (cm^2) is the effective area of the electrode and ΔV (V) represents the potential drop during discharge.

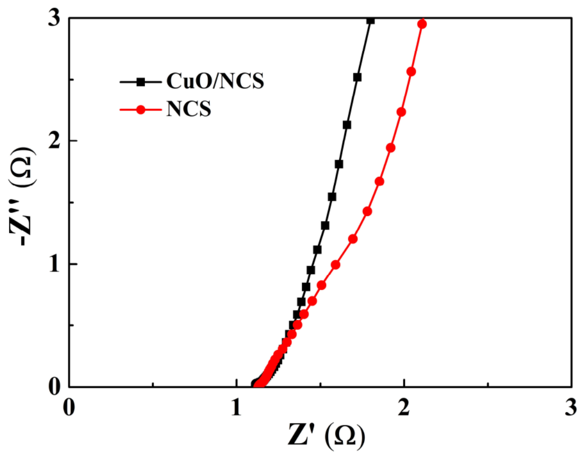

Figure S2. EIS spectra for CuO/NCS and NCS electrodes.
